# Crystal structure of a second triclinic polymorph of 2-methyl­pyridinium picrate

**DOI:** 10.1107/S205698901501912X

**Published:** 2015-10-17

**Authors:** Jeganathan Gomathi, Doraisamyraja Kalaivani

**Affiliations:** a PG and Research Department of Chemistry, Seethalakshmi Ramaswami College, Tiruchirappalli 620 002, Tamil Nadu, India

**Keywords:** crystal structure, polymorphism, 2-methyl­pyridinium picrate, 3-methyl­pyridinium picrate, 2,4,6-tri­nitro­phenolate

## Abstract

The title mol­ecular salt, C_6_H_8_N^+^·C_6_H_2_N_3_O_7_^−^ (systematic name: 2-methyl­pyridinium 2,4,6-tri­nitro­phenolate), crystallizes with two cations and two anions in the asymmetric unit. In the crystal, the cations are linked to the anions *via* bifurcated N—H⋯(O,O) hydrogen bonds, generating *R*_1_^2^(6) graph-set motifs. Numerous C—H⋯O hydrogen bonds are observed between these cation–anion pairs, which result in a three-dimensional network. In addition, weak aromatic π–π stacking between the 2-methyl­pyridinium rings [inter-centroid distance = 3.8334 (19) Å] and very weak stacking [inter-centroid distance = 4.0281 (16) Å] between inversion-related pairs of picrate anions is observed. The title salt is a second triclinic polymorph of the structure (also with *Z*′ = 2) reported earlier [Anita *et al.* (2006). *Acta Cryst.* C**62**, o567–o570; Chan *et al.* (2014 ▸). *CrystEngComm*, **16**, 4508–4538]. In the title compound, the cations and anions display a chequerboard arrangement when viewed down [100], whereas in the first polymorph, (010) layers of alternating cations and anions are apparent in a [100] view. It is inter­esting that the unit-cell lengths are almost identical for the two polymorphs, although the inter-axial angles are quite different.

## Related literature   

For the first triclinic polymorph of 2-methyl­pyridinium picrate, see: Anitha *et al.* (2006 ▸); Chan *et al.* (2014 ▸). For the crystal structure of the isomeric 3-methyl­pyridinium picrate, see: Gomathi & Kalaivani (2015 ▸).
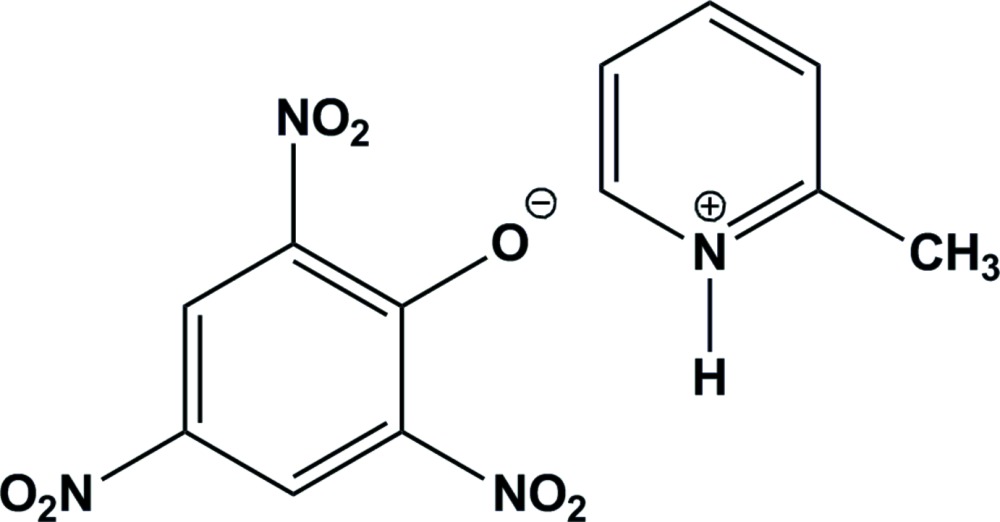


## Experimental   

### Crystal data  

C_6_H_8_N^+^·C_6_H_2_N_3_O_7_^−^
*M* *_r_* = 322.24
Triclinic, 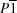

*a* = 8.1524 (4) Å
*b* = 11.8809 (6) Å
*c* = 14.6377 (9) Å
α = 102.077 (3)°
β = 90.001 (3)°
γ = 100.692 (3)°
*V* = 1361.21 (13) Å^3^
*Z* = 4
Mo *K*α radiation
μ = 0.13 mm^−1^
*T* = 296 K
0.35 × 0.35 × 0.30 mm

### Data collection  

Bruker Kappa APEXII CCD diffractometer
Absorption correction: multi-scan (*SADABS*; Bruker, 2004 ▸) *T*_min_ = 0.952, *T*_max_ = 0.969
25854 measured reflections
4789 independent reflections
3165 reflections with *I* > 2σ(*I*)
*R*_int_ = 0.034

### Refinement  

*R*[*F* ^2^ > 2σ(*F* ^2^)] = 0.052
*wR*(*F* ^2^) = 0.159
*S* = 1.06
4789 reflections
423 parameters
H atoms treated by a mixture of independent and constrained refinement
Δρ_max_ = 0.35 e Å^−3^
Δρ_min_ = −0.27 e Å^−3^

### 

Data collection: *APEX2* (Bruker, 2004 ▸); cell refinement: *SAINT* (Bruker, 2004 ▸); data reduction: *SAINT*; program(s) used to solve structure: *SIR92* (Altomare *et al.*, 1993 ▸); program(s) used to refine structure: *SHELXL2014* (Sheldrick, 2015 ▸); molecular graphics: *ORTEP-3 for Windows* (Farrugia, 2012 ▸) and *Mercury* (Macrae *et al.*, 2008 ▸); software used to prepare material for publication: *SHELXL2014*.

## Supplementary Material

Crystal structure: contains datablock(s) global, I. DOI: 10.1107/S205698901501912X/hb7512sup1.cif

Structure factors: contains datablock(s) I. DOI: 10.1107/S205698901501912X/hb7512Isup2.hkl

Supporting information file. DOI: 10.1107/S205698901501912X/hb7512Isup3.cml

**Fig. 1.**
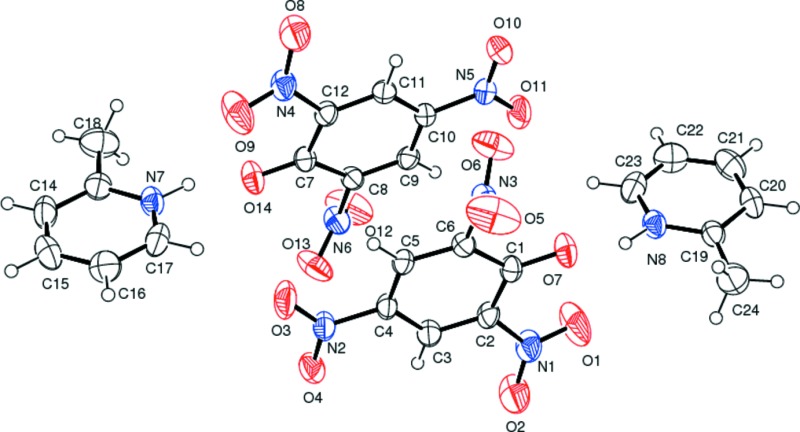
ORTEP view of the title molecular salt with displacement ellipsoids drawn at 40% probability.

ORTEP . DOI: 10.1107/S205698901501912X/hb7512fig1.tif
*ORTEP* view of the title mol­ecular salt with displacement ellipsoids drawn at 40% probability.

**Fig. 2.**
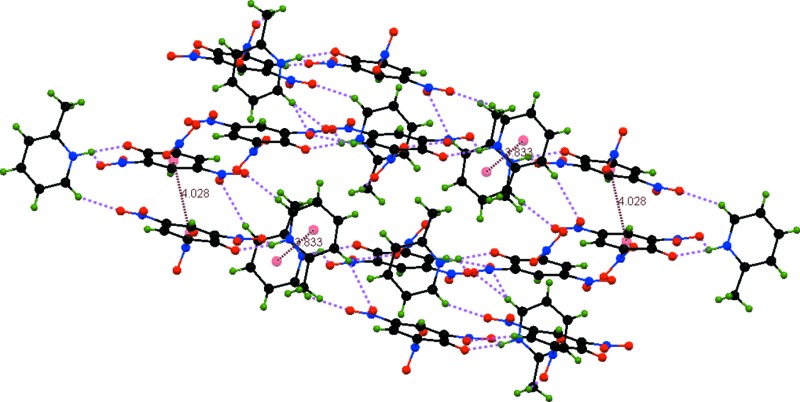
A partial view of the crystal packing diagram of the title molecular salt (hydrogen bonds and π–π stacking are shown as dotted lines).

. DOI: 10.1107/S205698901501912X/hb7512fig2.tif
 A partial view of the crystal packing diagram of the title mol­ecular salt (hydrogen bonds and π–π stacking are shown as dotted lines).

CCDC reference: 1417625

Additional supporting information:  crystallographic information; 3D view; checkCIF report

## Figures and Tables

**Table 1:** Hydrogen-bond geometry (, )

*D*H*A*	*D*H	H*A*	*D* *A*	*D*H*A*
N7H7*A*O9	0.95(4)	2.28(4)	2.813(4)	114(3)
N7H7*A*O14	0.95(4)	1.76(4)	2.678(3)	160(3)
N8H8*A*O1	0.94(4)	2.35(4)	2.894(4)	117(3)
N8H8*A*O7	0.94(4)	1.76(4)	2.660(3)	158(4)
C5H5O2^i^	0.93	2.50	3.423(4)	170
C9H9O8^ii^	0.93	2.45	3.365(3)	167
C14H14O10^iii^	0.93	2.54	3.456(4)	167
C17H17O3	0.93	2.34	3.078(4)	136
C18H18*B*O12^i^	0.96	2.64	3.488(5)	148
C20H20O13^iv^	0.93	2.55	3.247(4)	132
C23H23O8^ii^	0.93	2.63	3.394(4)	140
C23H23O11	0.93	2.36	3.122(4)	139

Symmetry codes: (i) 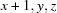; (ii) 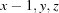; (iii) 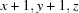; (iv) 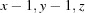.

## supplementary crystallographic information

### S1. Comment

Previous attempt in our laboratories to synthesize carbon-bonded anionic sigma complex with two heterocyclic moieties (substituted imidazole and pyridine) from the ethanolic solution containing 2-chloro-1,3,5-trinitrobenzene, hydantoin and 3-methylpyridine yielded 3-methylpyridinium picrate, a triclinic polymorph (Gomathi & Kalaivani, 2015). In the present work, a similar attempt with 2-methylpyridine instead of 3-methylpyridine in the reaction mixture, yielded 2-methylpyridinium picrate which crystallizes in the triclinic system with space group P1. Fig. 1 & 2 depict ORTEP and packing view of title molecular salt of present investigation respectively. Anita et al. (Anitha et al., 2006) have synthesized 2-methylpyridinium picrate by slow evaporation of the aqueous solution containing pyridoxine and picric acid in a 1:1 stoichiometric ratio at room temperature. They isolated instead of the expected picric acid complex with pyridoxine, crystals of 2-methylpyridinium picrate. Another group (Chan et al., 2014) has prepared 2-methylpyridinium picrate by adding picric acid to liquid 2-methylpyridine without other organic solvents. 2-Methylpyridinium picrate synthesized by both the groups also crystallize in the triclinic system with space group P1. The unit cell parameters of 2-methylpyridinium picrate of both the groups are nearly similar. However, 2-methypyridinium picrate reported in this article differs in the inter-axial bond angles noticeably. In addition to this observation, no disorder is observed in the title molecule, whereas, 2-methylpyridinium picrate reported by Anita et al. one of the oxygen atoms of the nitro group of picrate anion is disordered, with occupancy factors of 0.71 and 0.29. The dihedral angles between the planes of phenyl ring of picrate anions and that of 2-methylpyridinium cations of two molecules present in the asymmetric unit are greater than 80 ° [dihedral angle between (i) planes constituting C1-C2-C3-C4-C5-C6 and N7-C13-C14-C15-C16-C17, 85.54 (11)°; (ii) C1-C2-C3-C4-C5-C6 and N8-C19-C20-C21-C22-C23, 87.60 (11)°; (iii) C7-C8-C9-C10-C11-C12 and N7-C13-C14-C15-C16-C17, 80.60 (11)°; (iv) C7-C8-C9-C10-C11-C12 and N8-C19-C20-C21-C22-C23, 82.49 (10)°], which unambigously reflects the absence of π-bonding between the aromatic rings of anion and cation and supports the fact that the main contributing factor of the formation of the product is proton-transfer reaction. Protonation of the nitrogen atom is further evidenced from the values of the C-N bond distances. N-H···O hydrogen bonding is noticed between the cation and anion parts of two molecules of asymmetric unit and the bifurcation at N-H forming N-H···O hydrogen bonds with the oxygen atoms of phenolate and nitro group results in R_1_^2^(6) ring motif and this sort of linkage is highly responsible for the stability of the molecule. Along with this ring motif, other ring motifs such as R^2^_2_(7), R^3^_3_(13) and R^4^_3_(19) are also stabilizing the crystal system. The nitro group involved in forming R_1_^2^(6) ring motif bends only slightly from the plane of the aromatic ring to which it is attached [dihedral angles, 21.68 (16)° and 24.16 (12)°], whereas, the other nitro group lying on the other side of C-O^-^ bond twists from the ring remarkably [dihedral angles, 79.94 (12)° and 53.29 (15)°]. This kind of twisting may probably reduce the strain due to overcrowding around C-O^-^. The plane of the nitro group para with respect to C-O^-^ lies almost in the plane of the phenyl ring [dihedral angles, 5.02 (19)° and 3.08 (29)°].

### S2. Experimental

2-Chloro-1,3,5-trinitrobenzene [2.56 g (0.01 mol)] was dissolved in 30 ml of rectified spirit and mixed with hydantoin [1.00 g (0.01 mol)] in 20 ml of the same solvent. After mixing of these two solutions, 3 ml of 2-methylpyridine (0.03 mol) was added and the solution was heated to 318 K. The solution was stirred at this temperature with the help of magnetic stirrer for 5 h. The solution was cooled to room temperature and then filtered carefully. The clear maroon-red colour solution obtained was allowed to evaporate slowly maintaining the temperature at 293 K. After a period of six weeks, maroon-red coloured crystals formed from the solution. The crystals were filtered, powdered and washed with 30 ml of dry ether and recrystallized from rectified spirit. Instead of the expected carbon-bonded anionic sigma complex with hydantoin, crystals of 2-methylpyridinium picrate were obtained (yield: 70%; m.p.: 423 K).

### S3. Refinement

Crystal data, data collection and structure refinement details are summarized.

### Crystal data

**Table:**

C_6_H_8_N^+^·C_6_H_2_N_3_O_7_^−^	*Z* = 4
*M**_r_* = 322.24	*F*(000) = 664
Triclinic, *P*1	*D*_x_ = 1.572 Mg m^−^^3^
*a* = 8.1524 (4) Å	Mo *K*α radiation, λ = 0.71073 Å
*b* = 11.8809 (6) Å	Cell parameters from 6819 reflections
*c* = 14.6377 (9) Å	θ = 2.5–25.5°
α = 102.077 (3)°	µ = 0.13 mm^−^^1^
β = 90.001 (3)°	*T* = 296 K
γ = 100.692 (3)°	Block, yellow
*V* = 1361.21 (13) Å^3^	0.35 × 0.35 × 0.30 mm

### Data collection

**Table:**

Bruker Kappa APEXII CCD diffractometer	4789 independent reflections
Radiation source: fine-focus sealed tube	3165 reflections with *I* > 2σ(*I*)
Graphite monochromator	*R*_int_ = 0.034
ω and φ scan	θ_max_ = 25.0°, θ_min_ = 2.0°
Absorption correction: multi-scan (*SADABS*; Bruker, 2004)	*h* = −9→9
*T*_min_ = 0.952, *T*_max_ = 0.969	*k* = −14→14
25854 measured reflections	*l* = −17→17

### Refinement

**Table:**

Refinement on *F*^2^	0 restraints
Least-squares matrix: full	Hydrogen site location: mixed
*R*[*F*^2^ > 2σ(*F*^2^)] = 0.052	H atoms treated by a mixture of independent and constrained refinement
*wR*(*F*^2^) = 0.159	*w* = 1/[σ^2^(*F*_o_^2^) + (0.0576*P*)^2^ + 1.224*P*] where *P* = (*F*_o_^2^ + 2*F*_c_^2^)/3
*S* = 1.06	(Δ/σ)_max_ < 0.001
4789 reflections	Δρ_max_ = 0.35 e Å^−^^3^
423 parameters	Δρ_min_ = −0.27 e Å^−^^3^

### Special details

**Table:**

Geometry. All e.s.d.'s (except the e.s.d. in the dihedral angle between two l.s. planes) are estimated using the full covariance matrix. The cell e.s.d.'s are taken into account individually in the estimation of e.s.d.'s in distances, angles and torsion angles; correlations between e.s.d.'s in cell parameters are only used when they are defined by crystal symmetry. An approximate (isotropic) treatment of cell e.s.d.'s is used for estimating e.s.d.'s involving l.s. planes.

### Fractional atomic coordinates and isotropic or equivalent isotropic displacement parameters (Å^2^)

**Table:**

	*x*	*y*	*z*	*U*_iso_*/*U*_eq_	
C1	0.2716 (3)	0.1874 (2)	0.86637 (19)	0.0366 (6)	
C2	0.2025 (3)	0.2910 (2)	0.8744 (2)	0.0393 (7)	
C3	0.2970 (3)	0.4009 (2)	0.8818 (2)	0.0411 (7)	
H3	0.2457	0.4657	0.8877	0.049*	
C4	0.4672 (3)	0.4148 (2)	0.88062 (19)	0.0369 (6)	
C5	0.5471 (3)	0.3206 (2)	0.87618 (19)	0.0359 (6)	
H5	0.6630	0.3308	0.8777	0.043*	
C6	0.4504 (3)	0.2134 (2)	0.86961 (19)	0.0345 (6)	
C7	0.6949 (3)	0.3848 (2)	0.64217 (19)	0.0367 (6)	
C8	0.5153 (3)	0.3557 (2)	0.62748 (19)	0.0359 (6)	
C9	0.4225 (3)	0.2467 (2)	0.61397 (19)	0.0372 (6)	
H9	0.3071	0.2346	0.6047	0.045*	
C10	0.5014 (3)	0.1533 (2)	0.61406 (19)	0.0361 (6)	
C11	0.6715 (3)	0.1706 (2)	0.62624 (19)	0.0382 (7)	
H11	0.7239	0.1071	0.6248	0.046*	
C12	0.7637 (3)	0.2815 (2)	0.64042 (19)	0.0370 (6)	
C13	1.1475 (4)	0.6975 (3)	0.6877 (2)	0.0460 (7)	
C14	1.2631 (4)	0.7880 (3)	0.7391 (3)	0.0559 (9)	
H14	1.3302	0.8401	0.7090	0.067*	
C15	1.2802 (4)	0.8019 (3)	0.8328 (3)	0.0592 (9)	
H15	1.3584	0.8635	0.8667	0.071*	
C16	1.1837 (4)	0.7263 (3)	0.8779 (3)	0.0572 (9)	
H16	1.1961	0.7346	0.9422	0.069*	
C17	1.0688 (4)	0.6385 (3)	0.8267 (3)	0.0535 (8)	
H17	1.0012	0.5860	0.8562	0.064*	
C18	1.1222 (5)	0.6719 (4)	0.5849 (3)	0.0747 (11)	
H18A	1.1979	0.7286	0.5597	0.112*	
H18B	1.1430	0.5948	0.5592	0.112*	
H18C	1.0093	0.6755	0.5691	0.112*	
C19	−0.1835 (3)	−0.1337 (2)	0.8099 (2)	0.0412 (7)	
C20	−0.2865 (4)	−0.2209 (3)	0.7485 (2)	0.0524 (8)	
H20	−0.3591	−0.2782	0.7708	0.063*	
C21	−0.2835 (4)	−0.2242 (3)	0.6552 (3)	0.0627 (10)	
H21	−0.3535	−0.2839	0.6138	0.075*	
C22	−0.1775 (5)	−0.1400 (3)	0.6221 (3)	0.0640 (10)	
H22	−0.1753	−0.1408	0.5584	0.077*	
C23	−0.0760 (4)	−0.0555 (3)	0.6837 (3)	0.0569 (9)	
H23	−0.0024	0.0022	0.6623	0.068*	
C24	−0.1811 (5)	−0.1215 (3)	0.9123 (2)	0.0641 (9)	
H24A	−0.2608	−0.1842	0.9281	0.096*	
H24B	−0.0715	−0.1247	0.9343	0.096*	
H24C	−0.2096	−0.0477	0.9412	0.096*	
N1	0.0244 (3)	0.2838 (3)	0.8789 (2)	0.0583 (8)	
N2	0.5652 (3)	0.5307 (2)	0.88834 (18)	0.0459 (6)	
N3	0.5319 (3)	0.1137 (2)	0.86915 (19)	0.0420 (6)	
N4	0.9426 (3)	0.2915 (2)	0.6504 (2)	0.0513 (7)	
N5	0.4056 (3)	0.0360 (2)	0.59800 (18)	0.0463 (6)	
N6	0.4285 (3)	0.4513 (2)	0.6237 (2)	0.0485 (7)	
N7	1.0518 (3)	0.6267 (2)	0.7348 (2)	0.0458 (6)	
N8	−0.0805 (3)	−0.0542 (2)	0.7744 (2)	0.0455 (6)	
O1	−0.0686 (3)	0.1928 (2)	0.8495 (3)	0.1149 (13)	
O2	−0.0287 (3)	0.3707 (2)	0.9134 (3)	0.0944 (10)	
O3	0.7155 (3)	0.5408 (2)	0.8829 (2)	0.0747 (8)	
O4	0.4947 (3)	0.61454 (18)	0.90051 (17)	0.0594 (6)	
O5	0.5817 (4)	0.1009 (3)	0.9423 (2)	0.0988 (11)	
O6	0.5417 (4)	0.0464 (2)	0.79711 (19)	0.0767 (8)	
O7	0.1934 (2)	0.08461 (17)	0.86122 (15)	0.0512 (6)	
O8	1.0032 (3)	0.2064 (2)	0.6182 (2)	0.0866 (9)	
O9	1.0276 (3)	0.3819 (2)	0.6908 (2)	0.0953 (10)	
O10	0.4784 (3)	−0.04584 (18)	0.59417 (15)	0.0536 (6)	
O11	0.2538 (3)	0.0224 (2)	0.5862 (2)	0.0737 (8)	
O12	0.3364 (4)	0.4393 (3)	0.5559 (2)	0.0983 (11)	
O13	0.4468 (3)	0.5344 (2)	0.6878 (2)	0.0752 (8)	
O14	0.7744 (2)	0.48653 (17)	0.64961 (16)	0.0523 (6)	
H7A	0.970 (5)	0.565 (3)	0.700 (2)	0.072 (11)*	
H8A	−0.003 (5)	0.005 (3)	0.814 (3)	0.085 (12)*	

### Atomic displacement parameters (Å^2^)

**Table:**

	*U*^11^	*U*^22^	*U*^33^	*U*^12^	*U*^13^	*U*^23^
C1	0.0308 (14)	0.0384 (16)	0.0384 (16)	−0.0002 (12)	−0.0031 (11)	0.0091 (13)
C2	0.0229 (13)	0.0440 (17)	0.0499 (18)	0.0025 (12)	−0.0006 (11)	0.0104 (14)
C3	0.0349 (15)	0.0379 (16)	0.0528 (19)	0.0088 (12)	0.0022 (13)	0.0132 (14)
C4	0.0305 (14)	0.0343 (15)	0.0437 (17)	−0.0009 (11)	0.0022 (12)	0.0098 (13)
C5	0.0269 (13)	0.0381 (15)	0.0403 (16)	0.0011 (11)	0.0017 (11)	0.0074 (12)
C6	0.0314 (14)	0.0352 (15)	0.0366 (16)	0.0059 (11)	−0.0018 (11)	0.0071 (12)
C7	0.0318 (14)	0.0365 (16)	0.0390 (16)	0.0017 (12)	−0.0023 (11)	0.0063 (13)
C8	0.0317 (14)	0.0326 (15)	0.0423 (17)	0.0067 (11)	−0.0019 (11)	0.0052 (12)
C9	0.0268 (13)	0.0414 (16)	0.0409 (17)	0.0048 (12)	0.0012 (11)	0.0046 (13)
C10	0.0329 (14)	0.0318 (15)	0.0411 (17)	0.0019 (11)	0.0042 (11)	0.0057 (12)
C11	0.0361 (15)	0.0349 (15)	0.0450 (17)	0.0097 (12)	0.0056 (12)	0.0094 (13)
C12	0.0274 (13)	0.0402 (16)	0.0428 (17)	0.0048 (12)	−0.0006 (11)	0.0086 (13)
C13	0.0430 (16)	0.0397 (17)	0.060 (2)	0.0148 (14)	0.0040 (14)	0.0156 (15)
C14	0.0464 (18)	0.0427 (18)	0.079 (3)	−0.0026 (14)	0.0071 (17)	0.0229 (17)
C15	0.053 (2)	0.0429 (19)	0.074 (3)	−0.0037 (15)	−0.0136 (17)	0.0069 (17)
C16	0.0511 (19)	0.063 (2)	0.057 (2)	0.0121 (17)	−0.0037 (16)	0.0115 (18)
C17	0.0365 (16)	0.056 (2)	0.072 (3)	0.0031 (14)	0.0095 (15)	0.0270 (18)
C18	0.098 (3)	0.076 (3)	0.057 (2)	0.035 (2)	0.006 (2)	0.014 (2)
C19	0.0365 (15)	0.0333 (15)	0.0545 (19)	0.0067 (12)	0.0012 (13)	0.0106 (14)
C20	0.0489 (18)	0.0364 (17)	0.067 (2)	−0.0052 (14)	−0.0052 (16)	0.0111 (16)
C21	0.063 (2)	0.048 (2)	0.069 (3)	0.0044 (17)	−0.0196 (18)	−0.0004 (18)
C22	0.071 (2)	0.074 (3)	0.050 (2)	0.022 (2)	−0.0042 (18)	0.0120 (19)
C23	0.0455 (18)	0.062 (2)	0.071 (3)	0.0114 (16)	0.0139 (17)	0.0315 (19)
C24	0.073 (2)	0.063 (2)	0.055 (2)	0.0104 (18)	0.0016 (17)	0.0122 (18)
N1	0.0306 (14)	0.0527 (17)	0.091 (2)	0.0057 (13)	0.0026 (13)	0.0164 (16)
N2	0.0393 (14)	0.0389 (15)	0.0577 (17)	−0.0015 (11)	0.0034 (11)	0.0140 (12)
N3	0.0369 (13)	0.0374 (14)	0.0507 (17)	0.0042 (10)	−0.0032 (11)	0.0095 (13)
N4	0.0319 (13)	0.0450 (16)	0.078 (2)	0.0065 (12)	−0.0019 (12)	0.0159 (14)
N5	0.0416 (15)	0.0393 (15)	0.0535 (16)	−0.0004 (12)	0.0106 (11)	0.0073 (12)
N6	0.0413 (14)	0.0418 (15)	0.0621 (18)	0.0093 (11)	−0.0072 (13)	0.0090 (14)
N7	0.0325 (13)	0.0381 (14)	0.0644 (19)	0.0009 (11)	−0.0052 (12)	0.0107 (13)
N8	0.0334 (13)	0.0389 (14)	0.0624 (19)	0.0024 (11)	−0.0012 (12)	0.0103 (13)
O1	0.0293 (13)	0.0564 (17)	0.239 (4)	−0.0057 (12)	−0.0048 (17)	−0.003 (2)
O2	0.0400 (14)	0.0627 (17)	0.181 (3)	0.0171 (12)	0.0187 (16)	0.0202 (19)
O3	0.0350 (13)	0.0545 (15)	0.131 (2)	−0.0054 (10)	0.0125 (13)	0.0251 (15)
O4	0.0605 (14)	0.0346 (12)	0.0830 (17)	0.0070 (10)	0.0091 (12)	0.0143 (11)
O5	0.151 (3)	0.096 (2)	0.0671 (19)	0.072 (2)	−0.0282 (18)	0.0147 (16)
O6	0.108 (2)	0.0571 (16)	0.0671 (18)	0.0379 (15)	0.0000 (15)	−0.0016 (14)
O7	0.0380 (11)	0.0385 (12)	0.0740 (15)	−0.0062 (9)	−0.0104 (10)	0.0170 (10)
O8	0.0375 (13)	0.0568 (16)	0.165 (3)	0.0165 (12)	0.0103 (15)	0.0163 (17)
O9	0.0410 (14)	0.0557 (16)	0.176 (3)	0.0013 (12)	−0.0321 (16)	0.0013 (18)
O10	0.0617 (14)	0.0335 (12)	0.0650 (15)	0.0068 (10)	0.0075 (11)	0.0113 (10)
O11	0.0383 (13)	0.0528 (14)	0.120 (2)	−0.0063 (10)	0.0119 (13)	0.0102 (14)
O12	0.122 (2)	0.091 (2)	0.089 (2)	0.0606 (19)	−0.0448 (19)	0.0001 (16)
O13	0.0726 (17)	0.0431 (14)	0.101 (2)	0.0219 (12)	−0.0229 (14)	−0.0149 (14)
O14	0.0399 (11)	0.0373 (12)	0.0764 (16)	−0.0041 (9)	−0.0136 (10)	0.0151 (11)

### Geometric parameters (Å, º)

**Table:**

C1—O7	1.256 (3)	C17—N7	1.328 (4)
C1—C2	1.429 (4)	C17—H17	0.9300
C1—C6	1.431 (4)	C18—H18A	0.9600
C2—C3	1.371 (4)	C18—H18B	0.9600
C2—N1	1.441 (3)	C18—H18C	0.9600
C3—C4	1.367 (4)	C19—N8	1.334 (4)
C3—H3	0.9300	C19—C20	1.369 (4)
C4—C5	1.385 (4)	C19—C24	1.475 (4)
C4—N2	1.441 (3)	C20—C21	1.358 (5)
C5—C6	1.353 (4)	C20—H20	0.9300
C5—H5	0.9300	C21—C22	1.366 (5)
C6—N3	1.459 (3)	C21—H21	0.9300
C7—O14	1.244 (3)	C22—C23	1.348 (5)
C7—C12	1.437 (4)	C22—H22	0.9300
C7—C8	1.446 (4)	C23—N8	1.325 (4)
C8—C9	1.348 (4)	C23—H23	0.9300
C8—N6	1.455 (4)	C24—H24A	0.9600
C9—C10	1.382 (4)	C24—H24B	0.9600
C9—H9	0.9300	C24—H24C	0.9600
C10—C11	1.370 (4)	N1—O1	1.196 (3)
C10—N5	1.438 (3)	N1—O2	1.206 (3)
C11—C12	1.365 (4)	N2—O3	1.213 (3)
C11—H11	0.9300	N2—O4	1.222 (3)
C12—N4	1.446 (3)	N3—O5	1.193 (3)
C13—N7	1.337 (4)	N3—O6	1.195 (3)
C13—C14	1.377 (4)	N4—O9	1.200 (3)
C13—C18	1.478 (5)	N4—O8	1.215 (3)
C14—C15	1.351 (5)	N5—O10	1.222 (3)
C14—H14	0.9300	N5—O11	1.225 (3)
C15—C16	1.358 (5)	N6—O13	1.198 (3)
C15—H15	0.9300	N6—O12	1.213 (3)
C16—C17	1.356 (5)	N7—H7A	0.95 (4)
C16—H16	0.9300	N8—H8A	0.94 (4)
			
O7—C1—C2	127.2 (2)	C13—C18—H18A	109.5
O7—C1—C6	121.0 (3)	C13—C18—H18B	109.5
C2—C1—C6	111.7 (2)	H18A—C18—H18B	109.5
C3—C2—C1	123.7 (2)	C13—C18—H18C	109.5
C3—C2—N1	116.3 (3)	H18A—C18—H18C	109.5
C1—C2—N1	120.0 (2)	H18B—C18—H18C	109.5
C4—C3—C2	119.5 (3)	N8—C19—C20	117.5 (3)
C4—C3—H3	120.2	N8—C19—C24	118.3 (3)
C2—C3—H3	120.2	C20—C19—C24	124.2 (3)
C3—C4—C5	121.4 (2)	C21—C20—C19	120.5 (3)
C3—C4—N2	119.0 (2)	C21—C20—H20	119.8
C5—C4—N2	119.5 (2)	C19—C20—H20	119.8
C6—C5—C4	117.6 (2)	C20—C21—C22	120.0 (3)
C6—C5—H5	121.2	C20—C21—H21	120.0
C4—C5—H5	121.2	C22—C21—H21	120.0
C5—C6—C1	126.0 (3)	C23—C22—C21	118.6 (3)
C5—C6—N3	118.6 (2)	C23—C22—H22	120.7
C1—C6—N3	115.4 (2)	C21—C22—H22	120.7
O14—C7—C12	126.6 (2)	N8—C23—C22	120.4 (3)
O14—C7—C8	122.2 (2)	N8—C23—H23	119.8
C12—C7—C8	111.0 (2)	C22—C23—H23	119.8
C9—C8—C7	125.3 (2)	C19—C24—H24A	109.5
C9—C8—N6	117.4 (2)	C19—C24—H24B	109.5
C7—C8—N6	117.3 (2)	H24A—C24—H24B	109.5
C8—C9—C10	119.0 (2)	C19—C24—H24C	109.5
C8—C9—H9	120.5	H24A—C24—H24C	109.5
C10—C9—H9	120.5	H24B—C24—H24C	109.5
C11—C10—C9	120.7 (2)	O1—N1—O2	120.9 (3)
C11—C10—N5	119.2 (2)	O1—N1—C2	120.3 (3)
C9—C10—N5	120.1 (2)	O2—N1—C2	118.9 (3)
C12—C11—C10	119.6 (3)	O3—N2—O4	122.7 (2)
C12—C11—H11	120.2	O3—N2—C4	118.2 (2)
C10—C11—H11	120.2	O4—N2—C4	119.1 (2)
C11—C12—C7	124.4 (2)	O5—N3—O6	122.5 (3)
C11—C12—N4	116.0 (2)	O5—N3—C6	117.9 (3)
C7—C12—N4	119.6 (2)	O6—N3—C6	119.6 (3)
N7—C13—C14	117.2 (3)	O9—N4—O8	121.5 (3)
N7—C13—C18	117.6 (3)	O9—N4—C12	120.1 (3)
C14—C13—C18	125.2 (3)	O8—N4—C12	118.4 (3)
C15—C14—C13	120.7 (3)	O10—N5—O11	122.8 (2)
C15—C14—H14	119.7	O10—N5—C10	119.1 (2)
C13—C14—H14	119.7	O11—N5—C10	118.1 (2)
C14—C15—C16	120.4 (3)	O13—N6—O12	123.8 (3)
C14—C15—H15	119.8	O13—N6—C8	119.2 (3)
C16—C15—H15	119.8	O12—N6—C8	116.9 (3)
C17—C16—C15	118.3 (3)	C17—N7—C13	122.7 (3)
C17—C16—H16	120.8	C17—N7—H7A	119 (2)
C15—C16—H16	120.8	C13—N7—H7A	118 (2)
N7—C17—C16	120.6 (3)	C23—N8—C19	123.0 (3)
N7—C17—H17	119.7	C23—N8—H8A	116 (2)
C16—C17—H17	119.7	C19—N8—H8A	121 (2)
			
O7—C1—C2—C3	178.5 (3)	C15—C16—C17—N7	0.2 (5)
C6—C1—C2—C3	1.6 (4)	N8—C19—C20—C21	−0.7 (5)
O7—C1—C2—N1	1.0 (5)	C24—C19—C20—C21	178.5 (3)
C6—C1—C2—N1	−175.8 (3)	C19—C20—C21—C22	−0.2 (5)
C1—C2—C3—C4	0.8 (5)	C20—C21—C22—C23	0.8 (5)
N1—C2—C3—C4	178.3 (3)	C21—C22—C23—N8	−0.5 (5)
C2—C3—C4—C5	−2.9 (4)	C3—C2—N1—O1	160.5 (4)
C2—C3—C4—N2	179.9 (3)	C1—C2—N1—O1	−21.9 (5)
C3—C4—C5—C6	2.4 (4)	C3—C2—N1—O2	−20.0 (5)
N2—C4—C5—C6	179.6 (2)	C1—C2—N1—O2	157.6 (3)
C4—C5—C6—C1	0.3 (4)	C3—C4—N2—O3	−176.7 (3)
C4—C5—C6—N3	−177.2 (2)	C5—C4—N2—O3	6.0 (4)
O7—C1—C6—C5	−179.2 (3)	C3—C4—N2—O4	3.7 (4)
C2—C1—C6—C5	−2.1 (4)	C5—C4—N2—O4	−173.6 (3)
O7—C1—C6—N3	−1.6 (4)	C5—C6—N3—O5	79.3 (4)
C2—C1—C6—N3	175.5 (2)	C1—C6—N3—O5	−98.5 (3)
O14—C7—C8—C9	176.3 (3)	C5—C6—N3—O6	−103.1 (3)
C12—C7—C8—C9	0.1 (4)	C1—C6—N3—O6	79.1 (3)
O14—C7—C8—N6	−1.4 (4)	C11—C12—N4—O9	−156.7 (3)
C12—C7—C8—N6	−177.6 (2)	C7—C12—N4—O9	25.7 (4)
C7—C8—C9—C10	0.2 (4)	C11—C12—N4—O8	22.4 (4)
N6—C8—C9—C10	177.9 (3)	C7—C12—N4—O8	−155.2 (3)
C8—C9—C10—C11	−1.0 (4)	C11—C10—N5—O10	−1.1 (4)
C8—C9—C10—N5	−178.8 (3)	C9—C10—N5—O10	176.7 (3)
C9—C10—C11—C12	1.4 (4)	C11—C10—N5—O11	−179.2 (3)
N5—C10—C11—C12	179.2 (2)	C9—C10—N5—O11	−1.4 (4)
C10—C11—C12—C7	−1.1 (4)	C9—C8—N6—O13	126.6 (3)
C10—C11—C12—N4	−178.6 (3)	C7—C8—N6—O13	−55.5 (4)
O14—C7—C12—C11	−175.7 (3)	C9—C8—N6—O12	−51.1 (4)
C8—C7—C12—C11	0.3 (4)	C7—C8—N6—O12	126.8 (3)
O14—C7—C12—N4	1.7 (4)	C16—C17—N7—C13	1.6 (5)
C8—C7—C12—N4	177.8 (2)	C14—C13—N7—C17	−2.4 (4)
N7—C13—C14—C15	1.4 (5)	C18—C13—N7—C17	177.0 (3)
C18—C13—C14—C15	−177.9 (3)	C22—C23—N8—C19	−0.4 (5)
C13—C14—C15—C16	0.2 (5)	C20—C19—N8—C23	1.0 (4)
C14—C15—C16—C17	−1.1 (5)	C24—C19—N8—C23	−178.2 (3)

### Hydrogen-bond geometry (Å, º)

**Table:**

*D*—H···*A*	*D*—H	H···*A*	*D*···*A*	*D*—H···*A*
N7—H7*A*···O9	0.95 (4)	2.28 (4)	2.813 (4)	114 (3)
N7—H7*A*···O14	0.95 (4)	1.76 (4)	2.678 (3)	160 (3)
N8—H8*A*···O1	0.94 (4)	2.35 (4)	2.894 (4)	117 (3)
N8—H8*A*···O7	0.94 (4)	1.76 (4)	2.660 (3)	158 (4)
C5—H5···O2^i^	0.93	2.50	3.423 (4)	170
C9—H9···O8^ii^	0.93	2.45	3.365 (3)	167
C14—H14···O10^iii^	0.93	2.54	3.456 (4)	167
C17—H17···O3	0.93	2.34	3.078 (4)	136
C18—H18*B*···O12^i^	0.96	2.64	3.488 (5)	148
C20—H20···O13^iv^	0.93	2.55	3.247 (4)	132
C23—H23···O8^ii^	0.93	2.63	3.394 (4)	140
C23—H23···O11	0.93	2.36	3.122 (4)	139

Symmetry codes: (i) *x*+1, *y*, *z*; (ii) *x*−1, *y*, *z*; (iii) *x*+1, *y*+1, *z*; (iv) *x*−1, *y*−1, *z*.
