# Supplementary figures and images for: Crystal structure of a second triclinic polymorph of 2-methyl­pyridinium picrate
Source: Acta Crystallogr E Crystallogr Commun. 2015 Oct 17;71(Pt 11):o848–9. doi: 10.1107/S205698901501912X (PMC4645006; doi:10.1107/S205698901501912X)

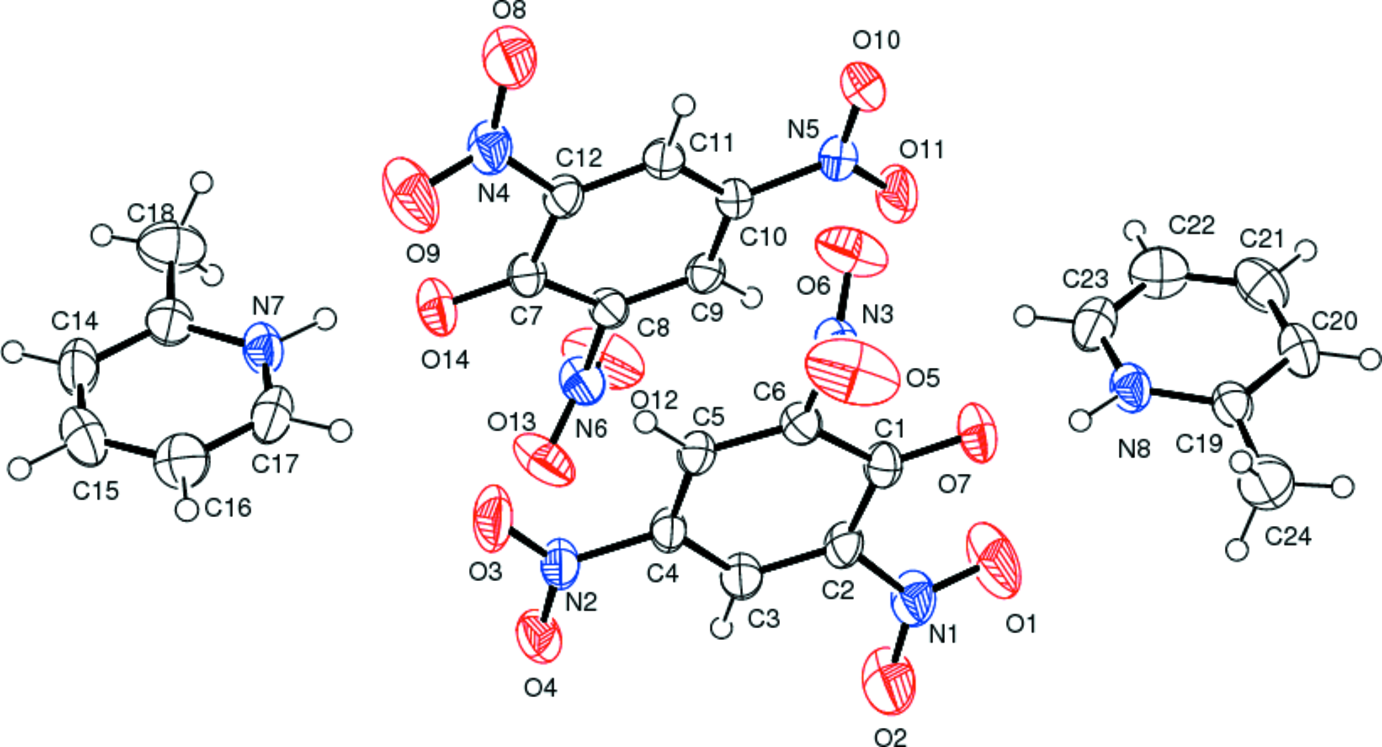

Supplement: Supplementary file 4 [file e-71-0o848-fig1.tif]

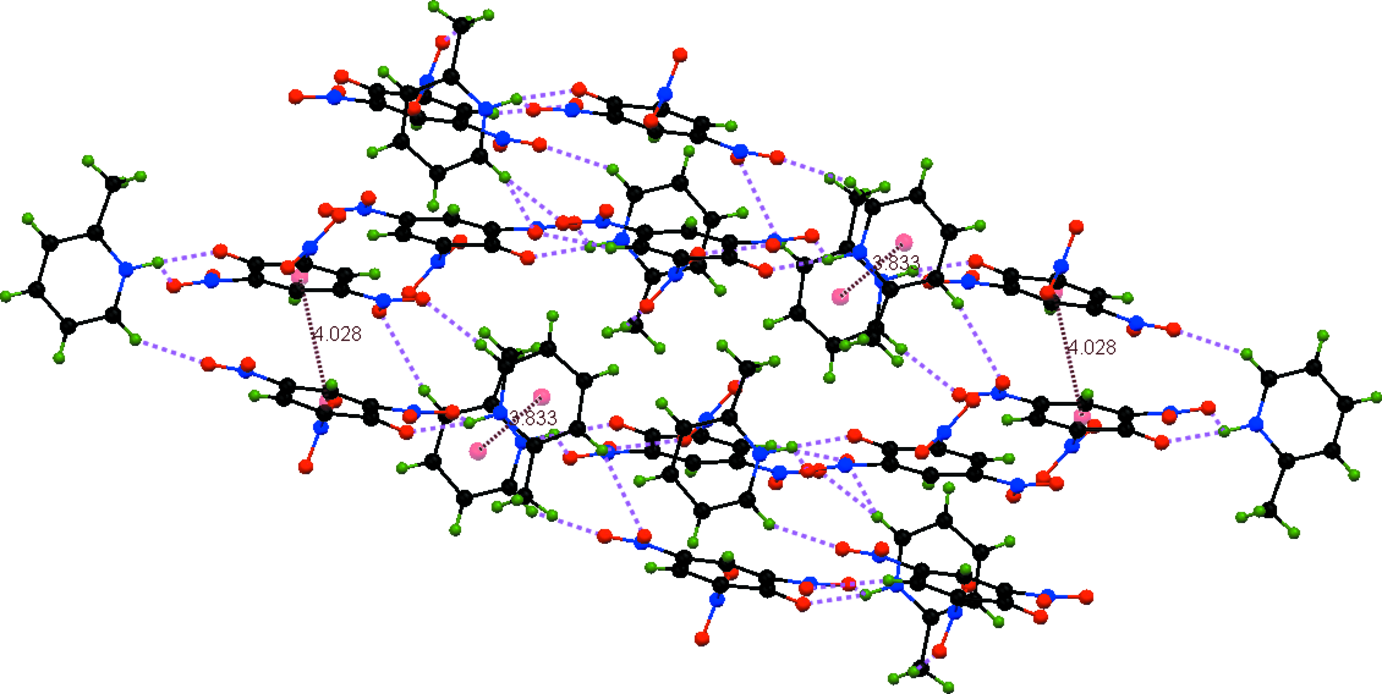

Supplement: Supplementary file 5 [file e-71-0o848-fig2.tif]
